# Fixed Airflow Obstruction in Asthma: A Problem of the Whole Lung Not of Just the Airways

**DOI:** 10.3389/fphys.2022.898208

**Published:** 2022-05-23

**Authors:** Sandra Rutting, Cindy Thamrin, Troy J. Cross, Gregory G. King, Katrina O. Tonga

**Affiliations:** ^1^ Airway Physiology and Imaging Group, The Woolcock Institute of Medical Research, The University of Sydney, Sydney, NSW, Australia; ^2^ The Department of Respiratory Medicine, Royal North Shore Hospital, Sydney, NSW, Australia; ^3^ Faculty of Medicine and Health, The University of Sydney, Sydney, NSW, Australia; ^4^ The Department of Thoracic and Transplant Medicine, St Vincent’s Hospital, Sydney, NSW, Australia; ^5^ St Vincent’s Healthcare Clinical Campus, School of Clinical Medicine, UNSW Medicine and Health, University of New South Wales Sydney, Sydney, NSW, Australia

**Keywords:** asthma, fixed airflow obstruction, airway remodeling, parenchymal remodeling, lung elastic recoil

## Abstract

Abstract Asthma with irreversible or fixed airflow obstruction (FAO) is a severe clinical phenotype that is difficult to treat and is associated with an accelerated decline in lung function and excess morbidity. There are no current treatments to reverse or prevent this excessive decline in lung function in these patients, due to a lack of understanding of the underlying pathophysiology. The current paradigm is that FAO in asthma is due to airway remodeling driven by chronic inflammation. However, emerging evidence indicates significant and critical structural and functional changes to the lung parenchyma and its lung elastic properties in asthma with FAO, suggesting that FAO is a ‘whole lung’ problem and not just of the airways. In this Perspective we draw upon what is known thus far on the pathophysiological mechanisms contributing to FAO in asthma, and focus on recent advances and future directions. We propose the view that structural and functional changes in parenchymal tissue, are just as (if not more) important than airway remodeling in causing persistent lung function decline in asthma. We believe this paradigm of FAO should be considered when developing novel treatments.

## Introduction

Asthma with irreversible or fixed airflow obstruction (FAO) is a clinical phenotype that is underappreciated and, subsequently, undertreated because our understanding of this phenotype is limited. Asthma with FAO is often (mis)labelled as chronic obstructive pulmonary disease (COPD) yet may develop despite nil-to-negligible smoking history or exposure to noxious agents. The prevalence of FAO is higher in those patients with severe asthma, being most frequent in older patients with long standing history of the condition (Lee et al., 2007). There is no current treatment to reverse or prevent excessive lung function decline in these severe asthmatics with FAO, due to a lack of understanding the underlying pathophysiology. The current paradigm is that FAO in asthma is due to airway remodeling driven by persistent eosinophilic inflammation. Treatment with standard asthma therapy, such as inhaled corticosteroids and bronchodilators, likely prevents excessive lung function loss in some patients, although there is little specific evidence to support this, likely due to difficulty in long term studies with a primary outcome of FEV1 decline. In many however, standard treatment does not prevent FAO from developing suggesting other mechanisms may play a role in the pathophysiology of this condition. The heavy focus on airway remodeling as the cause of FAO in asthma overlooks the potentially important contributions from disease-related changes in the mechanical properties of the lung parenchyma. Our group and others have recently shown that in asthma with FAO, there are also critical and substantial structural and functional changes to the lung parenchyma and its lung elastic properties. There is now a large body of evidence showing that loss of lung elasticity and even alveolar destruction, which results in mild alveolar dilation and reduced radial traction and mechanical support of airways, is an important mechanism causing FAO. In this Perspective we draw upon what is known thus far on the pathophysiological mechanisms contributing to FAO in asthma. We propose that structural and functional changes in lung parenchymal tissue are just as (if not more) important than airway remodeling in causing persistent lung function decline in asthma with FAO.

### Definition of Fixed Airflow Obstruction

There is no standard definition of fixed or persistent airflow obstruction, despite irreversibility in asthma being recognised for decades (Turner-Warwick, 1977). This lack of definition may be due to the absence of a clear consensus on what constitutes reversibility of airflow obstruction in asthma. Is it the progressive loss of bronchodilator responsiveness? Or is it a progressive deterioration of lung function to the point that airways obstruction persists despite a positive bronchodilator response (Eliasson & Degraff, 1985)? The most commonly used definition of FAO is a post-bronchodilator forced expiratory volume in the first second (FEV1)/forced vital capacity (FVC) ratio less than 0.7 or below the Lower Limit of Normal (LLN). This definition is different from a “lack of bronchodilator response” which may be defined as an increase in FEV1 and/or FVC of less than 12% or 200 ml compared to baseline values after inhalation of short-acting bronchodilator (Graham et al., 2019). It is estimated that about one-third of patients with FAO as defined by FEV1/FVC <0.7 still have a positive BD response (Hanania et al., 2021). Whether patients with and without BD reversibility represent two different subtypes of FAO with different underlying mechanisms and prognoses remains unclear.

Use of a fixed cut off of 0.7 lends itself to overestimation of disease in older people and underestimation in younger people (Quanjer et al., 2012). Preserved ratio impaired spirometry (PRISm) is a description applied to normal FEV1/FVC ratio but reduced FEV1 and may affect around 10% of smokers in a general population (Wan et al., 2018). However, it is non-specific and could represent either small lungs (perhaps due to reduced lung growth from early childhood), or could be due to gas trapping due to increased closure and flow limitation in small airways i.e. early small airways disease with only the latter being associated with poor clinical outcomes (Fortis et al., 2020).

### The Role of Airway Remodeling in FAO

Airway remodeling is currently thought to be the main mechanism by which FAO develops. Remodeling involves a wide array of pathophysiologic structural changes, including thickening of the reticular lamina, mucus gland hyperplasia, increased vascularity, and increased airway smooth muscle layer due to hyperplasia, hypertrophy and/or increased volume fraction of extracellular matrix (James & Wenzel, 2007). These changes subsequently lead to thickening of the airway wall and narrowing of the airway lumen, ultimately reducing airway distensibility. Airway remodeling has been demonstrated in mild-to-moderate asthma but is more pronounced in patients with severe disease (Carroll et al., 1993). There is no doubt regarding the role of airway remodeling in FAO; numerous computed tomography (CT) studies have shown that airway wall thickness is increased in patients with FAO compared with those without fixed obstruction, and several studies have demonstrated the adverse effects of airway remodeling on airflow obstruction and lung function in asthma (Ward et al., 2001; Ferreira et al., 2018; Smith et al., 2021).

### Structural Evidence for Parenchymal Remodeling

Structural parenchymal abnormalities in asthma were first demonstrated in post-mortem lungs of patients with severe asthma (Messer, Peters & Bennett, 1960; Mauad et al., 2004). Abnormalities included cleavage of terminal bronchiolar-alveolar tethering attachments with fragmented and decreased abnormal elastic fibres (Mauad et al., 2004), and localized parenchymal emphysema (Messer, Peters & Bennett, 1960). Gelb et al. later demonstrated mild, upper lobe predominant centrilobular emphysema with irregularly enlarged air spaces and fractured alveolar septa in post-mortem lungs of older lifelong non-smokers with asthma. The enlarged airspaces were found concurrent with more typical signs of airway remodeling of the large and small airways, including mucosal goblet cell metaplasia, thickening of both the basement membrane and airway smooth muscle layers (Gelb et al., 2014). In addition, areas of ‘senile lung’ such as alveolar duct ectasia, near homogenous alveolar hyperinflation and absence of tissue breakdown were also reported (Gelb et al., 2014), consistent with age-related changes (Verbeken et al., 1992). Within the same lungs, areas of normal tissue were also demonstrated, suggesting a heterogeneous distribution of parenchymal and airway abnormalities (Gelb et al., 2014). Computed Tomography (CT) of the lung allows non-invasive assessment of the morphological and structural properties of the airway and parenchyma. CT-studies have demonstrated areas of reduced lung density in chronic and severe asthma when compared to healthy controls (Biernacki et al., 1997; Mitsunobu et al., 2001; Busacker et al., 2009). Although reduced lung density (or percentage of low attenuation area; %LAA) is a widely used measurement of emphysema, it may also reflect an increase in alveolar size without alveolar wall destruction. The fractal dimension (FD) is another parameter that is derived from CT describing the structural complexity of airspaces, and reflects alveolar wall destruction causing coalescence of neighbouring airspaces (Mishima et al., 1999). A recent study by Shimizu et al. assessed airway and parenchymal remodeling using lung CT in severe asthma patients with and without FAO. Patients with FAO had more severe airway remodeling as shown by higher airway wall thickness and decreased airway intraluminal cross-sectional area compared to those without FAO. In addition, FD was lower and %LAA was higher in the patients with FAO compared to those without regardless of smoking status and asthma severity. Importantly, a decreased FD at baseline was independently associated with a greater longitudinal decline in FEV1 after 5 years in patients with severe asthma (Shimizu et al., 2021). This study provides strong evidence of FAO being a “whole lung” problem with involvement of both the parenchyma and airways in the pathophysiology of FAO and is the first paper linking structural parenchymal remodeling with future loss of lung function.

### Functional Consequences of Parenchymal Remodeling

Parenchymal tissue is the major determinant of elastic recoil in the lung, which helps to maintain the alveolar driving pressure and subsequently airway calibre during exhalation. The structural damage evident in the lung parenchyma in FAO may well lead to a loss of lung elastic recoil and, in turn, a reduction in the driving pressure during maximal expiratory flow and, therefore, reduced expiratory airflow. Moreover, with loss of alveolar attachments (Mauad et al., 2004) and fractured alveolar septa (Gelb et al., 2018), the airway-lung parenchyma interdependence is altered. The radial tethering force of the lung tissue to the airways, which holds the airways open, is reduced, leading to airway instability and airways that are prone to premature closure and collapse. Elastic recoil can be calculated from pressure-volume curves measured by an oesophageal balloon (Colebatch, Greaves & Ng, 1979) and several studies have demonstrated loss of elastic recoil in moderate-to-severe asthma (Gelb et al., 2002; Gelb et al., 2018; Tonga et al., 2020). Furthermore, a study of older non-smoking asthmatic adults with persistent maximal expiratory airflow limitation demonstrated a significant loss of lung elastic recoil, despite standard treatments, when compared with age-matched controls. In that study, the authors estimated that reduced elastic recoil accounted for up to 35–55% of the reduction in maximal flows at high lung volumes (Gelb & Zamel, 2000; Gelb et al., 2018). Similarly, Tonga et al. found that both increased lung compliance and loss of lung elastic recoil was common in older non-smokers with asthma and FAO. More importantly, it was demonstrated that increased lung compliance was related to more severe airflow obstruction as shown by reduced FEV1/FVC, and loss of elastic recoil was related to increased respiratory system resistance, i.e. reduced airway calibre as measured by oscillometry (Tonga et al., 2020). Also noteworthy was the observation in the same cohort that loss of lung elastic recoil was related to increased ventilation heterogeneity in diffusion-dependent airways (Sacin). This is further evidence that the mechanical properties of the lung parenchyma are also an important determinant of peripheral airway function in patients with asthma and FAO (Tonga et al., 2019).

### Contributions of Parenchymal vs. Airway Remodeling to the Development of FAO

**FIGURE 1:**
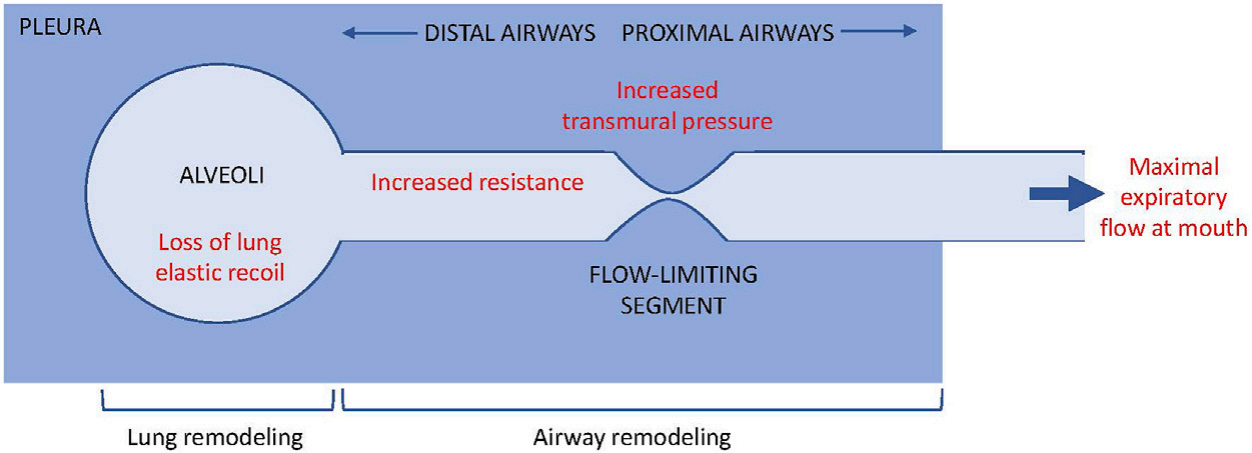
Waterflow model (Pride et al., 1967) and the contribution of airway vs. lung remodeling to flow limitation. During maximal expiratory flow limitation, air flow from the alveoli to the mouth becomes independent of the driving pressure or resistance downstream of the flow-limited segment, analogous to the independence of water flow from the height of a waterflow. The maximal flow at the mouth is determined by lung elastic recoil, transmural pressure across the flow-limited segment, and resistance upstream of the flow-limited segment.

In a classical paper, Pride et al. posited a ‘waterfall’ model to describe the determinants of maximal expiratory flow (Pride et al., 1967). We suggest that this model provides a useful framework to help understand how both airway and parenchymal remodeling may contribute to the development of FAO (Figure 1). Considering the airway as a tube with a collapsible (or flow-limiting) segment, Pride et al. showed that beyond a specific point along the tube, no changes in the downstream resistance (i.e. in the more central airways towards the mouth) or further increases in the driving pressure can produce any further increases in air flow, analogous to the independence of water flow in a waterfall on its height. The maximal flow generated is a function of lung elastic recoil, transmural pressure across the flow-limiting segment, and airway resistance upstream from the flow-limiting segment (i.e. in the more peripheral airways between the alveoli and the flow limiting segment). Furthermore, by comparing model versus empirical data from symptomatic smokers without a history of asthma, they proposed that bronchodilator responsive obstruction was due to the effects of airway smooth muscle tone on transmural pressure across the flow-limiting segment. In contrast, observations in irreversible obstruction were more consistent with increased upstream resistance (i.e. greater narrowing in the more peripheral airways), as well as with increased lung compliance or decreased lung elastic recoil with the latter thought to dominate. We suggest that in asthma with FAO, increased upstream resistance (very peripheral airways, close to the pressure source i.e. lung parenchyma) due to both airway remodeling and reduced lung elastic recoil must be present, as occurs in smoking-related COPD. Although the functional outcome might be similar between smoking-related COPD and asthma with FAO, the mechanisms and structural changes in the latter are unknown. There may be common pathophysiologic processes e.g. terminal airway obliteration but it is highly likely that there will be considerable differences as well e.g. severity of alveolar destruction, which means that to be able to prevent or reverse FAO we will require greater pathophysiologic understanding. The concept that presence of flow-limiting segments might be relevant in asthma due to airway remodeling, which may confer some degree of bronchodilator reversibility, since the authors of the aforementioned paper demonstrated in their model, the functional significance of bronchomotor tone on the flow-limiting segments. Bronchoconstriction typically produces a number of ventilation defects i.e. patchy airway closure (Farrow et al., 2012). Modelling (Tgavalekos et al., 2003) and experimental data (Farrow et al., 2012; Rutting et al., 2021) strongly implicate the combined effects of peripheral airway dysfunction (upstream resistance in the waterfall model) and airway closure (collapsibility and bronchoconstriction at the flow-limiting segment) in asthma. Ventilation defects on imaging were also related to FEV1 impairment and one could speculate that in asthma with FAO, the mechanical properties of the flow-limiting segments are then irreversibly impaired.

People with asthma are thought to have ‘stiffer’ airways as demonstrated by reduced airway distensibility (Brown et al., 2007; Kelly et al., 2013). Reduced airway distensibility in asthma does not increase following administration of a short acting bronchodilator (Brown et al., 2007), which suggests that airway distensibility is likely due to airway remodeling rather than an increase airway smooth muscle (ASM) contractility. Increased ASM thickness (Dunnill, Massarella & Anderson, 1969; Ebina et al., 1993) causes excessive airway narrowing during bronchoconstriction, despite normal muscle contractility. The net effect from increased ASM mass may be an increase in the load acting against the airway-distending forces therefore resulting in stiffer airways. This effect may be further exaggerated by any increase in the extracellular matrix within the smooth muscle layer. Stiffer airways could prevent dilation of airways following deep inspiration, but paradoxically stiffer airways may also resist narrowing.

ASM mechanics may also contribute to FAO, although this is only speculative based on mostly models from C. Seow et al. and J. Freberg et al. ASM is a dynamic organ that can adapt mechanically to its mechanical environment to optimise force generation and stiffness. The fluid or plastic nature of ASM, as it has been referred to, might facilitate ASM becoming stiffer when either the airway wall matrix becomes stiffer or the lung tissue becomes more compliant or its attachments to the outer wall of the airway become damaged. Any of these changes might then reduce the regular and rhythmic stretches from normal breathing, and intermittent larger stretches from regular sighs, which have proposed as being critical to maintaining compliance in ASM (Bosse et al., 2013).

Airway stiffness and progressive airway narrowing due to airway and/or parenchymal lung remodelling likely contribute to the decline in lung function in asthma (Lange et al., 1998), which declines more rapidly, in those with FAO (Ulrik & Backer, 1999).

### Proposed Mechanisms for Parenchymal Remodeling

**FIGURE 2:**
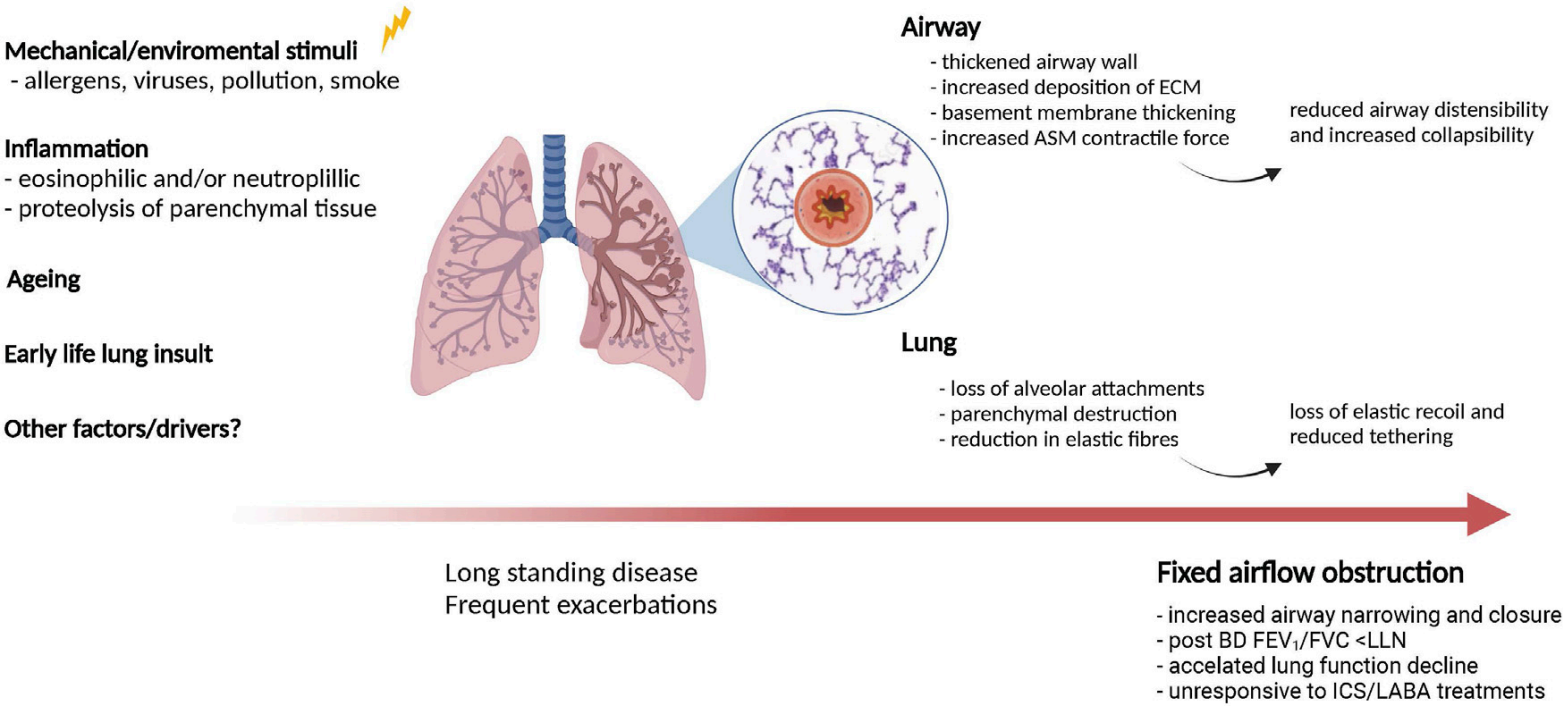
Potential mechanisms contributing to lung and airway remodelling in asthma with fixed airflow obstruction. Several factors and mechanisms may be involved in causing airway- and parenchymal remodeling in asthma patients with long-standing disease. Both of these processes likely contribute to fixed airflow obstruction in asthma.

While we have presented the functional significance of parenchymal changes in patients with asthma and FAO, the pathophysiologic mechanisms responsible for parenchymal remodeling and loss of lung elastic recoil in these patients are poorly understood. However, several factors and mechanisms may be involved in the pathogenesis of FAO in asthma (Figure 2).

### Inflammation

Activation of proteolytic cascades which affect parenchymal tissue have been proposed as an underlying mechanism for FAO (Gelb et al., 2018) and seems an obvious mechanism by which parenchymal damage occurs. Airway inflammation in asthma affects the full thickness of the airways and includes the outer airway wall and surrounding alveolar tissue (Kraft et al., 1996; Dolhnikoff et al., 2009). Proteolytic enzymes such as matrix metalloproteinases (MMPs), are increased in the airway wall and peribronchiolar parenchyma in fatal asthma (de Magalhaes Simoes et al., 2005; Dolhnikoff et al., 2009) and severe asthma (Andersson et al., 2011; Kuo et al., 2019). Circulating MMPs are also increased during acute exacerbations of asthma, and increased levels have been associated with more severe airflow obstruction (Oshita et al., 2003). What drives the likely degradation of matrix in the outer wall of airways and lung parenchyma, however, is very much unknown. Although uncontrolled eosinophilic inflammation has been a long-held paradigm and may be the predominant mechanism in some, evidence suggest that the drivers are heterogeneous. Early treatment with inhaled corticosteroids that reduces eosinophilic inflammation prevents loss of lung function in newly diagnosed asthma (Haahtela et al., 1994), but eosinophilic inflammation is inconsistently associated with FAO. Neutrophilic inflammation has also been associated with FAO development in several studies (Mogensen et al., 2020; Smith et al., 2021) and could represent a different FAO endotype. Smith et al. found at least two distinct endotypes of FAO; a group with persistent FAO characterized by neutrophilic sputum inflammation, remodelled airways and low but stable FEV1 and a group of patients who developed incident FAO that exhibited higher baseline sputum eosinophil content and a greater rate of FEV1 decline (Smith et al., 2021). Yet, other studies, including the study by Shimizu et al., found no relationships between eosinophilic- or neutrophilic markers and measurements of airway or parenchymal remodeling (Shimizu et al., 2021). Clearly, more studies are needed to better understand the role of inflammatory cells as well as granulocytes, in addition to the role of proteolytic mechanisms and their drivers, to parenchymal damage and FAO.

### Ageing

Fixed airflow obstruction is more common in older patients with asthma and thus the ageing process itself could be an additional or synergistic contributing factor. Tonga et al. demonstrated a negative correlation between FEV1/FVC and age in patients with FAO, even after accounting for age using z-scores (Tonga et al., 2019), suggesting an interaction between age and airflow obstruction. It is well known that lung function declines with age due to loss of lung elastic recoil and increasing lung compliance, secondary to reduction of the number of elastic fibres in the alveolar attachments and ducts and enlargement of airspaces (Turner, Mead & Wohl, 1968; Verbeken et al., 1992). Furthermore, the airways from older healthy lungs differ morphologically from those in young people; airway walls are nearly parallel and tapered smoothly in young people. Whereas compared to older lungs, occasional airways failed to taper and the airway wall may have irregularities (Wilson, Massarella & Pride, 1974). Furthermore, loss of terminal bronchioles demonstrated using a combination of *ex-vivo* conventional CT, whole lung micro-CT and micro-CT of extracted cores are an important structural component of age-related decline in lung function of healthy, non-smoking individuals (Verleden et al., 2021). Airway stiffness is also increased in older lungs compared to young healthy lungs (Wilson, Massarella & Pride, 1974), which suggests airway remodeling may also increase with age. These results suggests that the structural airway and lung tissue changes that occur with ageing could accentuate functional abnormalities seen in older people with asthma. However the contribution of the ageing process to FAO, is difficult to disentangle from the contribution of asthma itself.

### Early Loss of Lung Function

Epidemiological evidence suggest early deficits in lung function impaired lung growth and dysanapsis, developmental mismatch between airway and lung size (Smith et al., 2020; Smith et al., 2021), have a potential influence on lung function later in life. If initial loss of function and lung elasticity occurs at an early stage (McGeachie et al., 2016; Agusti & Faner, 2019), this lower starting point could eventually contribute to irreversible airflow obstruction later in life in a subset of patients. A study of asthma patients that were followed for over 20 years, showed that worse baseline lung function (FEV1) and less BD reversibility predicted the development of FAO later in life (Vonk et al., 2003). Interestingly, patients with FAO were found to be using less corticosteroids at follow up. Another longitudinal study of patients with childhood asthma demonstrated that patients with a reduced-growth lung pattern in childhood, as compared with those who had normal lung growth, had lower FEV1 values at enrolment, lower bronchodilator responses and greater airway hyperresponsiveness. Reduced lung function at baseline was the strongest predictor of longitudinal lung-function impairment (McGeachie et al., 2016). However, a study of non-smoking adults with asthma showed conflicting results with a higher degree of bronchodilator reversibility at baseline and long-term oral corticosteroid treatment being associated with FAO after 10 years of follow up. The risk factors identified in longitudinal studies are consistent with heterogeneous processes that lead to FAO, from possible *in utero* and all the way through childhood that is manifested in altered physiology (bronchodilator responsiveness and airway hyperresponsiveness) (Ulrik & Backer, 1999). Therefore, interactions between pathological processes and growth and maturation, might differ greatly to what happens with lung ageing.

### Additional Factors/Mechanisms

Several other factors including genetics, steroid use, air pollution, smoking exposure, frequent exacerbations, viral infections etc. that have been associated with loss of lung function (Int Panis et al., 2017) or FAO development, may further contribute to or modify inflammatory and remodeling processes in the airways and lungs of patients with asthma and FAO. The contribution of these factors are at present unknown. We suggest that to advance asthma treatment and to prevent fixed airflow obstruction, the underlying mechanisms of parenchymal (and airway) remodeling have to be understood, which goes far beyond the eosinophilic inflammation paradigm, because in doing so potential new treatment targets will be identified.

### How to Treat Fixed Airflow Obstruction?

While current main asthma treatments of inhaled corticosteroids and bronchodilators may have some benefit in asthma patients with FAO and allergic airway inflammation, they are not effective in preventing or reversing FAO. This is probably party due to a late diagnosis of FAO after irreversible damage/changes to the lungs and airways have already occurred, and partly because currently treatments are targeting the inflammatory process only. Newer biological treatments targeting allergic and eosinophilic inflammation also have limited effects; while omalizumab, an anti-IgE monoclonal antibody, reduced exacerbation rate it does not improve lung function in patients with FAO irrespective of bronchodilator (BD) reversibility (Hanania et al., 2021). Monoclonal antibodies targeting interleukin-5 or its receptor, reduce exacerbation rate and asthma symptoms, and have a variable benefit on FEV1 in patients with severe, uncontrolled eosinophilic asthma (Pavord et al., 2012; FitzGerald et al., 2016; FitzGerald et al., 2019). This was the case for patients with and without FAO (Chipps et al., 2020). However, these newer treatments are specifically targeting symptomatic asthmatics with corticosteroid resistant eosinophilic inflammation and although there is short term improvement, the effects on long term FEV1 decline are not known. There are currently no treatments available aimed directly at reducing airway- and/or parenchymal remodelling. Hence the need to establish specific mechanisms for FAO so we can develop novel treatments to treat and prevent FAO effectively.

## Conclusion

Altogether, the current data demonstrates that FAO is a ‘whole lung’ problem with strong evidence for structural and function changes to not only the airways but also the lung parenchyma causing FAO. This idea challenges the current concept of intrinsic airway narrowing resulting from airway remodeling as the sole cause of FAO in asthma. Current asthma treatments of inhaled corticosteroids and bronchodilators are not effective in preventing or reversing these lung abnormalities. Therefore, novel treatments are to be developed that need to consider this model of FAO. However, the underlying mechanisms for parenchymal remodeling in asthma remain poorly understood, as is the extent of its contribution to FAO relative to airway remodeling. As such, further asthma studies that focus on structure-function relationships, and the underlying cellular and molecular mechanisms that cause airway and parenchymal remodeling are needed to develop novel treatment strategies.

## Data Availability

The original contributions presented in the study are included in the article, further inquiries can be directed to the corresponding author.
